# Adsorption of Methylene Blue Dye by Cetyltrimethylammonium Bromide Intercalated Polyaniline-Functionalized Montmorillonite Clay Nanocomposite: Kinetics, Isotherms, and Mechanism Study

**DOI:** 10.3390/polym15173518

**Published:** 2023-08-23

**Authors:** Naima Mennas, Saadia Lahreche, Faiza Chouli, Lilia Sabantina, Abdelghani Benyoucef

**Affiliations:** 1 Water Science and Technology Laboratory, University of Mustapha Stambouli Mascara, Mascara 29000, Algeria; naimamennas.univ29@gmail.com; 2 Laboratory of Physico-Chemical Studies, University of Saïda, BP 138, Saïda 20000, Algeria; lahrechmg2010@gmail.com; 3 Faculty of Science and Technology, University of Mustapha Stambouli Mascara, Mascara 29000, Algeria; chouli.faiza@univ-mascara.dz; 4 Department of Clothing Technology and Manufacturing Engineering, Berlin University of Applied Sciences—HTW Berlin, 12459 Berlin, Germany

**Keywords:** montmorillonite, polyaniline, modification, adsorption, dye removal, wastewater

## Abstract

In this study, new adsorbents were prepared by modifying a montmorillonite clay (Mt) with cethyltrimethyl ammonium bromide (CTAB) to form CTAB-Mt, followed by a second modification process with polyaniline (PAni) to form PAni@CTAB-Mt by in situ polymerization of aniline. X-ray diffraction (XRD), X-ray fluorescence spectroscopy (XRF), X-ray photoelectron spectroscopy (XPS), thermogravimetric analysis (TGA), transmission electron microscopy (TEM), cyclic voltammetry (CV) and the Brunauer–Emmett–Teller (BET) technique were used to characterize the samples. These adsorbents were used in a batch process to remove methylene blue (MB) from aqueous solution. Factors investigated included initial pH of the solution, contact time and temperature. The adsorption data fit the Freundlich isotherm better than the Langmuir and Temkin isotherms. The maximum adsorption capacities (*q_eq_*) obtained were 108.82 mg·g^−1^, 71.20 mg·g^−1^ and 57.36 mg·g^−1^ for PAni@CTAB-Mt, CTAB-Mt and Mt, respectively. The enhanced adsorption capability of the hybrid material is due to increase in surface area and pore volume of the PAni@CTAB-Mt adsorbent. The adsorption results were found to fit well with the pseudo-second-order kinetics model, with highest correlation coefficient (*R*^2^) values of 0.954, 0.942 and 0.958 for Mt, CTAB-Mt and PAni@CTAB-Mt adsorbents, respectively. The pH and temperature had a significant effect on the adsorption process, and the negative values of ΔG suggest that the adsorption process was spontaneous and feasible. The desorption and reusability experiment indicated that PAni@CTAB-Mt has the potential to be a reusable adsorbent for MB removal.

## 1. Introduction

Water plays a vital role in all human activities. As many industrial effluents are discharged into the environment and freshwater resources are limited throughout the world, especially in developing countries, water and wastewater treatment seems to be the most effective strategy to deal with this problem [1,2,3,4]. Recently, many efforts have been made by researchers to purify water and wastewater. These include physical adsorption, separation, biodegradation, chemical degradation and integrated treatment [5,6]. Among all these efforts, physical adsorption plays a significant role. It offers several unique advantages, such as low energy consumption, fewer tools, environmental compatibility, ease of access and good efficiency. As a result of these excellent properties, physical adsorption offers a promising future [7,8,9,10].

Dyes are substances with an important application in various industries such as food, printing, plastics, textiles, paper, leather and pharmaceutical products. Methylene blue (C_16_H_18_ClN_3_S, MB) is a cationic dye that has been widely used in industry, such as in dyeing cotton, silk and wool [4]. It is a non-biodegradable and hazardous dye that is found in high concentrations in textile effluents [9], and it has a major impact on the health of water bodies and the photosynthesis of microorganisms in the aquatic environment [11]. Therefore, the treatment of effluent with MB dye before discharge is of interest due to its harmful effects on receiving waters [9,11].

Adsorption, with efficient adsorbent design as its core, is one of the most widely used techniques for dye removal and concentration from aqueous solution due to its low cost and simplicity of use [12]. Our work is dedicated to the preparation of novel modified montmorillonite (Mt). Mt is a cheap clay material with good performance in pollutants adsorption [12,13]. However, its development has been slowed down due to its low aqueous form dispersion, and it is susceptible to aggregation, which could affect the structural integrity, low affinity for dyes and difficulty in recovery after use [12,13,14,15]. Accordingly, in order to optimize the adsorption properties of Mt, the purified natural Mt was screened and used herein. In addition, cetyltrimethylammonium bromide (CTAB) could replace the cations in Mt to form organoclay, which can change the surface characteristics of Mt [12].

Polyaniline (PAni) as a conducting polymer has attracted much attention because of its scientific and technological interest because of its advantages such as redox property, high conductivity, higher environmental stability and easy preparation [11,12,13]. Moreover, it has a wide future application in water purification [12].

The incorporation of electroactive guest PAnis into host clays has attracted great attention due to their better processability, alongside their colloidal stability, mechanical strength and novel electrical and catalytic properties [12]. The main applications of PAni-Mt have been the corrosion protection of steel surfaces and the removal of various aqueous contaminants [12,13]. Several Mt-based materials have been prepared and used for dye adsorption. PANI/Fe_3_O_4_ composites were investigated for the removal of Basic blue 3 (BB3) dye from aqueous solution, and the highest adsorption capacity for (BB3) was obtained with capacities of 78.13 mg·g^−1^ [9]. Salah et al. synthesized polyaniline/glauconite nanocomposite for adsorption of Congo red dye from textile wastewater. It was reported that the adsorption capacity reached 14.1 mg·g^−1^ [11]. PAni/SiO_2_ composite was synthesized and applied for methylene blue removal. The equilibrium capacity was found to be 6.97 mg·g^−1^ [14]. A natural clay was used for the adsorption of Astrazon Red and Astrazon Blue. The adsorption capacity was found to be 47.6 mg·g^−1^ and 61.3 mg·g^−1^, respectively [16].

Some researchers suggested that CTAB could provide abundant active sites for dye removal from aqueous solution [12]. On the other hand, the polymer may have some different intercalation or adsorption properties in modified Mt. Motivated by this, an adsorbent ternary composite of PAni-modified CTAB@Mt was prepared to investigate its MB adsorption from aqueous solution. The characterizations, batch adsorption experiments and mechanism were carried out to study the MB adsorption behavior.

## 2. Materials and Methods

### 2.1. Materials

Aniline (C_6_H_5_NH_2_; Sigma Aldrich, Darmstadt, Germany. ≥99.5%), cethyltrimethyl ammonium bromide (CTAB; C_16_H_33_N(CH_3_)_3_Br; Merck, Darmstadt, Germany. ≥98%), ammonia solution (NH_4_OH; Merck, Riga, Lithuania. 25%), ammonium persulfate (APS; Merck, Riga, Lithuania. ≥98%), N-methylpyrrolidone (NMP; Merck, Darmstadt, Germany), sodium hydroxide (NaOH; Merck, Riga, Lithuania. 37%), hydrochloric acid (HCl; Merck, Riga, Lithuania. 75%), hydrogen peroxide (H_2_O_2_; Merck, Riga, Lithuania. 70%), sodium chloride (NaCl; Merck, Riga, Lithuania), Malachite Green (MG; C_23_H_25_ClN_2_; Merck, Darmstadt, Germany. 99%), ethanol (C_2_H_5_OH) (Merck, Darmstadt, Germany. 96%), filter paper and deionized water (DI) were used. Additionally, the montmorillonite clay (Mt) used in this study was obtained from ENOF (Maghnia, Tlemcen, Algeria).

### 2.2. Measurements

The surface morphology and elemental composition of the samples were studied by employing H7500-Hitachi (Tokyo, Japan) transmission electron microscopy (TEM). X-ray photoelectron spectroscopy (XPS) (3000 electron, AVG-Microtech-Multilab, Tokyo, Japan) was used to study the surface elemental composition of the samples. Thermogravimetric analysis (TGA) (Hitachi STA7200; Tokyo, Japan) was utilized to measure the thermal stability of the materials. A Hitachi-U3000 spectrophotometer (Tokyo, Japan) was used to study the optical properties and also to measure the equilibrium concentration of the dye at λ_max_ = 664 nm. FT-IR spectra of the synthesized samples were recorded using a Bruker Alpha spectrophotometer (Karlsruhe, Germany). X-ray fluorescence spectroscopy (XRF) was used by a Bruker AXS microanalyzer to determine the composition in weight percent (elemental wt %); the values were made in a helium gas purge. The surface area was determined from the adsorption isotherm by the Brunauer–Emmett–Teller (BET) method using an Autosorb-iQ instrument (Madrid, Spain). The materials were dried at 343 K for 24 h, and then pastilles were prepared using a FTIR mold [11,12,13]. The crystallinity properties of the samples were characterized by XRD (Bruker CCDApex, Madison, WI, USA) using *CuK_α_* radiation (*λ* = 1.5404 Å). The spacing value (*d*-spacing) is determined from the Bragg’s formula [12]:

$$d=\frac{\lambda }{2sin\theta }$$

where *λ*: the wavelength of X-rays; *θ*: the diffraction angle.

### 2.3. Preparation of PANI@CTAB-Mt Adsorbent

The Mt was intercalated according to a method reported by Cunha et al. [16]; 50 g of Mt was suspended in 600 mL of 1% CTAB solution under continuous stirring at 400 rpm for 24 h. To functionalize the CTAB-intercalated Mt (CTAB@Mt) surface, 30 mL of aniline monomer was added to the suspension under continuous stirring for 1 h. Then the APS (Ani:APS 1:1) as oxidant was added dropwise into the solution in an ice bath for 24 h under continuous stirring. Finally, the functionalized intercalated clay (PANI@CTAB-Mt) preparation was reached after filtration and rinsing several times with DI until the pH was close to neutral. The adsorbent was dried at 50 °C under vacuum conditions for 24 h.

### 2.4. Electrochemical Studies

These electrochemical properties of the materials were tested by cyclic voltammetry (CV), applying a conventional three-electrode electrochemical system [11,12,13].

### 2.5. Adsorption Tests

The adsorption capacity of MB in aqueous solution was evaluated in a batch experiment. Typically, an aqueous solution (25 mL, pH 2−12, time periods 5–180 min) containing MB in a concentration range of 10 to 200 mg·L^−1^ was incubated with a given amount of adsorbent at a temperature from 298 to 328 K. After the desired adsorption time, the adsorbents were separated by filtration. The MB concentration was analyzed by using a UV-visible spectrophotometer at 664 nm. The adsorption amount of the adsorbent for MB into was calculated according to equation [16]:

$$q_{eq}=\frac{C_{0}-C_{eq}}{w}$$

where $q_{eq}$ is the removal ratio (mg·g^−1^), $C_{0}$ is the initial metal ion concentration and $C_{eq}$ is the metal ion concentration at equilibrium (mg·L^−1^), and *w* is the weight of the adsorbent added (g·L^−1^).

The kinetic models of the MB were studied at 298 K for 4 h. The experimental results were investigated using the following models [12,13,16]:

$$\mathrm{Kinetic}\;\mathrm{model}\;\mathrm{of}\;\mathrm{pseudo}\;\mathrm{first}\;\mathrm{order}\;(\mathrm{PFO})\;\log(q_{eq}-q_{t})=\log q_{eq}-\frac{k_{1}}{2.303}t\;\mathrm{Kinetic}\;\mathrm{model}\;\mathrm{of}\;\mathrm{pseudo}\;\sec\mathrm{ond}\;\mathrm{order}\;(\mathrm{PSO})\;\frac{t}{q_{t}}=\frac{1}{k_{2}q_{eq}^{2}}+\frac{1}{q_{1}}t\;\mathrm{Kinetic}\;\mathrm{model}\;\mathrm{of}\;\mathrm{intraparticle}\;\mathrm{diffusion}\;(\mathrm{ID})\;q_{t}=k_{i}t^{0.5}+C$$

where $k_{1}$ is PFO rate constant (min^−1^); $k_{2}$ is PSO constant (g·mg^−1^.min^−1^); $q_{eq}$ is adsorption capacity at equilibrium (mg·g^−1^); $q_{t}$ is MB amount adsorbed at time t (mg·g^−1^); $k_{i}$ is intraparticle diffusion rate constant (g·mg^−1^·min^−1^); *C* is the boundary layer thickness.

The adsorption isotherms can be described as the process of mass transfer rate between the solid adsorbent and the adsorbate at constant temperature. Adsorption isotherms including Langmuir, Freundlich and Temkin were used to investigate the experimental data for adsorption using the following formulas [16,17,18]:

$$\mathrm{Langmuir}\;\mathrm{formula}:\;\frac{C_{eq}}{q_{eq}}=\frac{1}{K_{l}C_{m}}+\frac{C_{eq}}{q_{m}}\;\mathrm{Freundlich}\;\mathrm{formula}:\;lnq_{eq}=lnK_{f}+\frac{1}{n}lnC_{eq}\;\mathrm{Temkin}\;\mathrm{formula}:\;q_{eq}=Bln(K_{T}C_{eq})$$

where $K_{l}$ is the Langmuir constant (L·mg^−1^); $K_{f}$ is the Freudlich constant (mg^1–1/n^·g^−1^·L^1/n^); $K_{T}$ is the Temkin constant isotherm (L·mg^−1^); B is the Temkin constant of the adsorption heat (kJ·mol^−1^); $q_{m}$ is the maximum amount adsorbed (mg·g^−1^); $q_{eq}$ is the amount adsorbed at equilibrium (mg·g^−1^); $C_{eq}$ is the equilibrium concentration (mg·L^−1^); *n* is the heterogeneity factor.

The experimental results were applied for the evaluation of thermodynamics functions; namely, the entropy ($\Delta S^{0}$), standard enthalpy ($\Delta H^{0}$) and free energy ($\Delta G^{0}$) were evaluated from the equations [11,16]:

$$\Delta G^{0}=-RTlnK_{l}$$

$$\Delta G^{0}=\Delta H^{0}-T\Delta S^{0}$$

where the plot $(lnK_{l}$) versus $\frac{1}{T}$ was used to calculate the value of ($\Delta H^{0}$) and ($\Delta S^{0}$).

## 3. Results

### 3.1. Physicochemical Characteristics of the Samples

**Figure 1:**
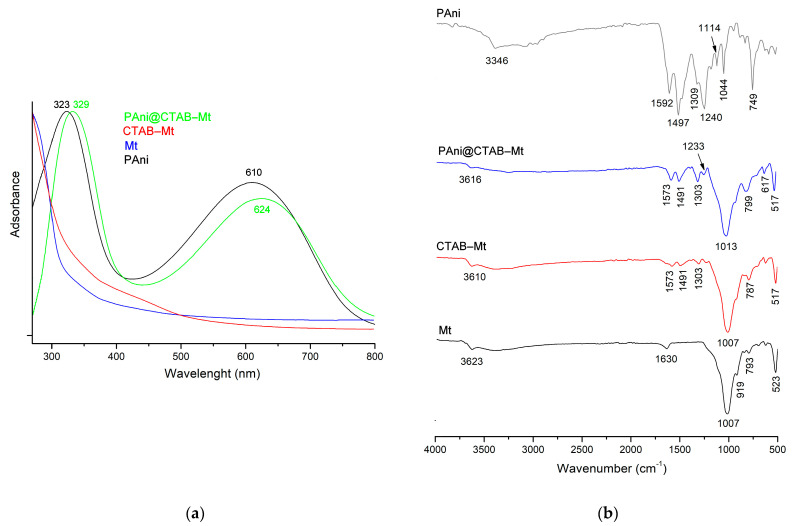
(**a**) UV-vis spectroscopy absorption spectra and (**b**) FTIR analysis of materials.

**Figure 2:**
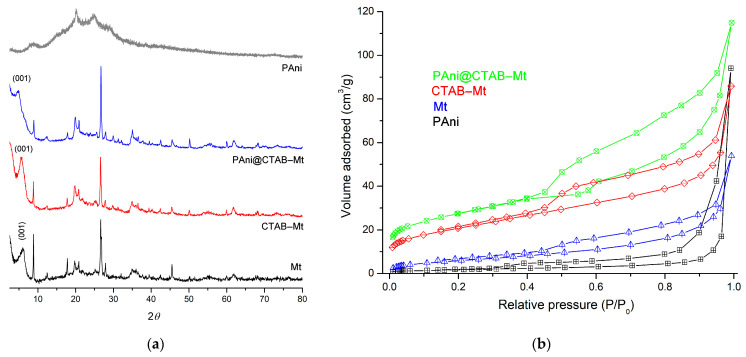
(**a**) X-ray diffraction (XRD) patterns and (**b**) nitrogen adsorption isotherms of samples.

To validate the chemical structures of the PAni, Mt, CTAB-Mt and PAni@CTAB-Mt, UV-vis, FTIR, XRD and XPS analysis were also conducted. The UV-vis spectrum (Figure 1a) of the PAni shows two obvious adsorptions at 323 and 610 nm, which are attributed to the π-π* transition in the benzenoid rings and π-polaron transition in the PAni chain [12], respectively. These characteristic bands of PAni are slightly red-shifted in the UV-vis spectrum of the PAni@CTAB-Mt, suggesting the interaction between PAni chain and CTAB-Mt (323 to 329 nm and 610 to 624 nm). In the FTIR spectrum of the PAni (Figure 2b), the bands located at 1592 and 1497 cm^−1^ should be attributed to the C=C stretching vibration of the quinonoid and benzene rings, respectively. The contraction peak at 1309 cm^−1^ corresponds to the C-N bond, while the stretching vibration of the -NH^+^= group and the bending vibration of the C-H bond are located at 1114 cm^−1^ and 749 cm^−1^, respectively. These results prove the chemical structures of typical PAni products [16]. The FTIR spectrum of the Mt shows that the band at 3623 cm^−1^ is related to the Al-O-H inter-octahedral [13]. The peaks observed at 1630 cm^−1^ suggest the possibility of water hydration in the Mt sample (H-O-H stretching) [14]. The band at 1007 cm^−1^ is a characteristic of layered silicate minerals and is attributed to the triple degenerate Si-O stretching ν3 (in-plane) vibration. In addition, the band located at 919 cm^−1^ is attributed to the deformation vibration of Al-OH-Al or Al-Al-OH [12]; and the band at 793 cm^−1^ is attributed to free silica or quartz admixtures, always present in natural Mt [12,13]. Furthermore, the band at 523 cm^−1^ is attributed to the deformation mode of the Al-O-Si function. Moreover, the comparison of the FTIR spectra of the Mt sample after modification by CTAB shows the appearance of new bands in the spectral range between 1300 cm^−1^ and 1575 cm^−1^. As for the PAni@CTAB-Mt sample, the 1007 cm^−1^ band (Si-O) of Mt is found to be shifted to 1013 cm^−1^, and the shift of the characteristic absorption bands of PAni is also observed.

The chemical composition of the studied samples was evaluated by XRF. From the data reported in Table 1, the predominance of aluminum can be observed, which is coherent with the trioctahedral character of the Mt minerals. The content of Fe, Na and K must be related to the presence of exchangeable cations. Moreover, the modification of the Mt with the CTAB and/or PAni shows higher organic matter content (CTAB-Mt 10.80 wt % and PAni@CTAB-Mt 14.06 wt %). The high organic matter values could be attributed to the formation of hybrid materials, which can lead to an increase in the adsorption capacity.

Figure 2a shows the X-ray diffraction patterns of Mt, CTAB-Mt, PAni@CTAB-Mt and PAni. Differences in crystallinity and mineralogical content of the starting samples can be detected between them. The pattern of the Mt sample showed sharp peaks, confirming the results of the montmorillonite clay analysis. The broad and intense peak at 2θ = 6.61° for the Mt sample corresponding to the (001) reflection in Mt modified by CTAB and PAni@CTAB shifts towards larger (d) spaces from 14.33 Å to 15.22 Å to 17.87 Å, respectively. The pattern of the PAni was characterized by lack of crystallinity. Since the conducting polymer material is in the mesoporous form, the formation of infinite non-uniform pores on the surface of the polymer was attributed to the amorphous nature of the product [16].

The textural characteristics of the prepared adsorbents were analyzed through N_2_ adsorption–desorption isotherm tests (Figure 2b). All the isotherms showed a type II isotherm with a distinctive type H3 hysteresis loop, suggesting the presence of a small macroporous component [12,17]. The pore size data width of the adsorbents is summarized in Table 2. The specific surface area (S_BET_) of Mt (91 m^2^·g^−1^) decreases after the modified surface and consists of 73 m^2^·g^−1^ for CTAB-Mt. Such reduction is determined by the practically complete filling of the micropores with CTAB molecules, resulting in the blocking of the access of nitrogen molecules to these pores. The PAni@CTAB-Mt adsorbent has an increased S_BET_ of 121 m^2^·g^−1^. This is due to the formation of a two-dimensional porous structure in the interlayer space of the PAni matrix.

**Figure 3:**
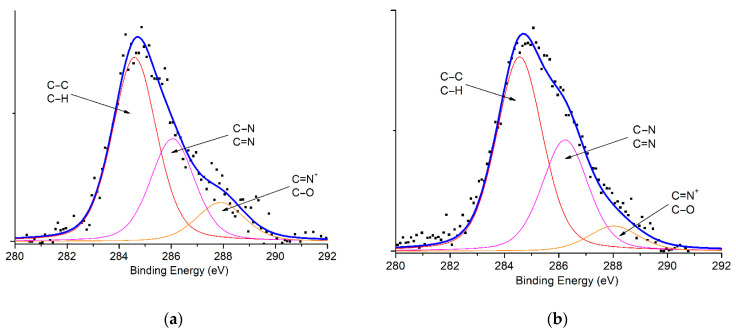
C1s scanning spectra of samples: (**a**) CTAB-Mt and (**b**) PAni@CTAB-Mt.

**Figure 4:**
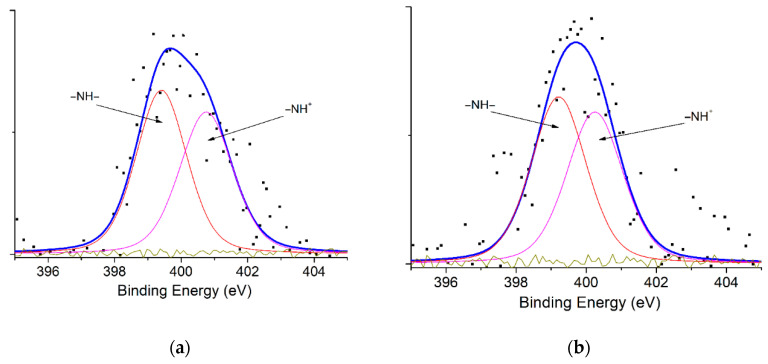
N1s scanning spectra of the samples: (**a**) CTAB-Mt and (**b**) PAni@CTAB-Mt.

XPS analysis was carried out to determine the surface elemental components (Figure 3 and Table 3). The atomic concentration of surface carbon increases with Mt modification by PAni and/or CTAB. Compared to Mt, the relative composition of aliphatic/aromatic carbon groups (C-C/C-H groups, ~284 eV) from CTAB-Mt and PAni@CTAB-Mt increases to 62.22% and 68.19%, respectively. Regarding the N1s spectrum (Figure 4), the fitted XPS spectra of CTAB-Mt showed two distinct peaks at 399.42 and 400.75 eV, corresponding to –NH– and =NH– groups, respectively. In addition, PAni@CTAB-Mt shows that the relative composition of –NH–/=NH– has become 56.89% and 43.11%, respectively.

**Figure 5:**
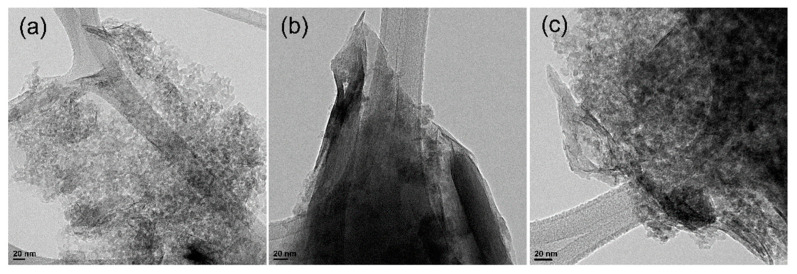
TEM images of (**a**) Mt, (**b**) CTAB-Mt and (**c**) PAni@CTAB-Mt.

In order to compare the differences in the textural structures of Mt and modified Mt and to see the effectiveness of the modification made, TEM images of the samples were taken and are shown in Figure 5. The TEM image in Figure 5a demonstrates the typical structure of the Mt image. It can be noticed from this image that there is a homogeneous distribution of the clay flakes. The TEM images of the CTAB-Mt in Figure 5b show a homogenous loading of the CTAB inside the Mt material. Comparison of the TEM micrographs in Figure 5a,b confirmed the formation of the CTAB-Mt sample. Moreover, the TEM image in Figure 5c depicts the distribution of the formed PAni assembled on CTAB within the interlayer spaces of Mt, which is compatible with the XRD and FTIR results in this investigation and confirms the formation of the Mt modified by PAni and/or CTAB.

**Figure 6:**
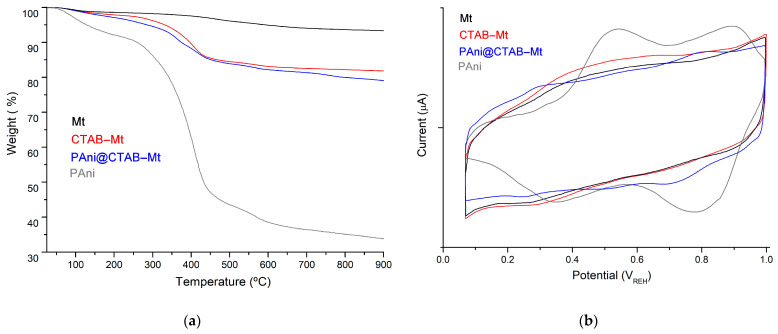
(**a**) TGA data of mass loss; (**b**) cyclic voltammogram response of materials.

Figure 6a shows the thermogravimetric analysis of the samples. Three distinct weight loss processes led to the thermal degradation of both PAni, CTAB-Mt and PAni@CTAB-Mt. The first weight loss below 130 °C is caused by the evaporation of the retained volatiles of the system and the adsorbed moisture. The elimination of oligomers took place in the second stage, which ran from 220 to 430 °C [12]. The second thermos, that leads to a variety of degradation products, is what causes the third stage to occur after 430 °C [13]. An increase in thermal stability was observed in the case of the CTAB-Mt (weight loss of 18.05%), which may be related to the effective integration of CTAB into the Mt structure. Compared to PAni@CTAB-Mt, it displayed a higher weight loss of 21.01% at 900 °C, which may be due to the fact that the CTAB-Mt contains PAni as a matrix in its interlayer. Moreover, the TGA curve data showed that above 220 °C the decomposition process of Mt starts and it exhibits a weight loss of 6.59% at 900 °C, whereas the weight loss of PAni is mainly concentrated between 290 °C and 530 °C, which is also related to the degradation of the PAni molecular chains [15].

Figure 6b shows cyclic voltammograms (CVs) of samples with potentials ranging from 0.05 V to 1.0 V and scanning rates of 50 mv·s^−1^. The results show that the CV curve of PAni@CTAB-Mt is clearly dissimilar from that of the tCTAB-Mt sample as a consequence of formation of PAni into the interlayer space. According to this result, the PAni matrix significantly affects the electrochemical behavior of PAni@CTAB-Mt, proving that it is more electrochemically active compared to the CTAB-Mt sample [18]. Conversely, the results for CTAB-Mt show that the CV curve was relatively comparable to the CV of the Mt sample. However, it displayed a sustained peak current, indicating that CTAB and Mt were fully electrochemically durable.

### 3.2. Adsorption Study of MB on Adsorbents

There are four factors involved in the adsorption of MB on adsorbents: the pH effect, the concentration effect, the kinetic effect and the temperature effect.

#### 3.2.1. pH Effect on MB Adsorption

**Figure 7:**
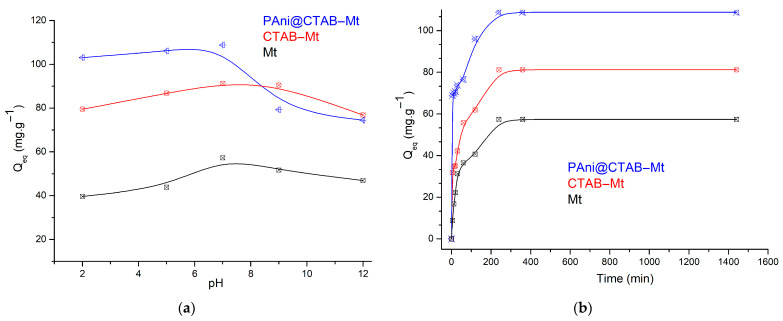
(**a**) Effect of pH on the adsorption capacity; (**b**) effect of contact time (C_0_: 150 mg·L^−1^; pH: 6.8; T: 298 K).

Variations in pH can alter the ionization degree of an adsorptive molecule as well as the surface properties of an adsorbent; therefore, the initial pH of a solution is a critical element in determining the capacity of an adsorbent in water treatment [19]. Dye solubility and adsorbent surface charge are both affected by pH. As a result, the adsorption efficiency changes as the pH changes. By varying the pH of the adsorbent–adsorbate solution from 2.0 to 12.0, the influence of pH on the adsorption mechanism of basic and acidic dyes was studied using different adsorbents. Amounts of 0.1 M NaOH or HCl were utilized to change the pH of the solution. Figure 7a depicts the results.

The highest adsorption capacity occurs at pH 6.8, at around 108 mg·g^−1^, but with increasing pH it decreases to 74 mg·g^−1^ at pH 12.0 (see Figure 7a). This result suggests that an acidic condition is more favorable for MB removal by PAni@CTAB-Mt. The higher adsorption capacity is due to the fact that the amino groups on the adsorbent are protonated at lower pH values, which makes the surface of PAni@CTAB-Mt positively charged and promotes electrostatic interaction between MB and the adsorbent, hence increasing adsorption capacity. In contrast, the surface of PAni@CTAB-Mt became less positively charged at higher pH values. The existence of OH^−^ in an alkaline environment produces a competitive impact with MB, resulting in a decrease in removal efficiency [20]. For CTAB-Mt, the adsorption capacity increases with increasing pH, until it reaches approximately 91 mg·g^−1^, then decreases to 76 mg·g^−1^ at pH 12.0. Moreover, the same phenomenon occurs when Mt adsorbent is used. Considering the small difference between the adsorption capacity at pH 6 and 7, the optimum pH value was chosen to be 6.8.

#### 3.2.2. Contact Time and Kinetic Modeling

Contact time helps to determine the rate at which harmful contaminants can be bound and be removed. In addition, contact time is a crucial factor in demonstrating the efficacy of an adsorbent in real-world use and in determining the best time for dye removal [15,16]. Batch adsorption studies were carried out at different contact times in the range of 5–1440 min to evaluate the impact of adsorbent and adsorbate contact time on the removal of MB using different adsorbents. Figure 7b depicts the results.

The analysis indicated that the adsorption of MB dye using different adsorbents was rapid in the first 30 min, then slowed down with time and reached equilibrium in 90 min for PAni@CTAB-Mt and 60 min for CTAB-Mt and Mt. With 100 min of shaking, the highest adsorption capacity values (108.82, 82.25 and 57.36 mg·g^−1^) of different adsorbents (PAni@CTAB-Mt, CTAB-Mt, and Mt) were obtained, respectively. As a result, the optimum contact time for maximum MB dye removal was determined to be 240 min for all adsorbents prepared. There was no significant change in the removal capacity after the optimum time.

It was noted that the adsorption of the MB was particularly quick during the first 60 min, indicating that the PAni matrices are very efficient in the removal process. This behavior may be explained by the large number of adsorption sites available at the beginning. Subsequently, the uptake process of MB slowed down and the equilibrium condition for the MB was reached in 240 min. The effect of the contact time of CTAB-Mt was investigated. During the first 60 min, its adsorption capacity increased dramatically. Thereafter, the percentage removal gradually increased before stabilizing for the next 120 min. In the early stages, a large number of adsorption sites on CTAB-Mt were rapidly occupied. In less than 60 min, the adsorption efficiency was nearly balanced.

The calculated parameters of the models are listed in Table 4. The coefficient of determination (*R*^2^) was used to compare the results obtained from the kinetic models and to determine the best model to describe MB adsorption on adsorbents. The strong *R*^2^ and the similarity between experimental and calculated q_eq_ values (mg·g^−1^) demonstrated the suitability of the PSO kinetic models to the kinetic data. Otherwise, the PFO model showed minimal suitability with a poor *R*^2^ for all materials used as adsorbents.

In order to investigate the mechanism controlling the kinetics of the dye adsorption process on the obtained materials, the ID model proposed by Weber-Morris [21] was used. It is possible to determine the mechanism of the adsorption process in a given system. This dependence can be single or multiple. In the case where the relationship is linear over the whole range and passes through the origin of the coordinate system, ID is the step that controls the adsorption process. On the other hand, if the linear relationship does not pass through the origin of the coordinate system, it means that ID is involved in the adsorption process, but it is not a speed-controlling step in the adsorption process. When the relationship is multilinear, the ID model mentions that there are two or more stages that make up and influence the adsorption process: The first (the fastest) stage is related to the external surface adsorption—the adsorbed molecules move from the solution to the surface of the adsorbent by diffusion through the boundary layer (diffusion in the boundary layer). The second stage includes the gradual diffusion of the adsorbate through the pores of the adsorbent (intraparticle diffusion). Finally, the third stage is an equilibrium state involving very slow diffusion of the adsorbate from the larger pores to the smaller ones (micropores) [21]. Similarly, the ID model data in Table 4 have a low regression coefficient (*R*^2^), and the C parameters have positive values and they are different from zero. Based on these considerations, it can be concluded that intraparticle diffusion affects the adsorption rate, but it is not the step that limits the whole process.

#### 3.2.3. Initial Concentration Effect and Isotherm Modeling

**Figure 8:**
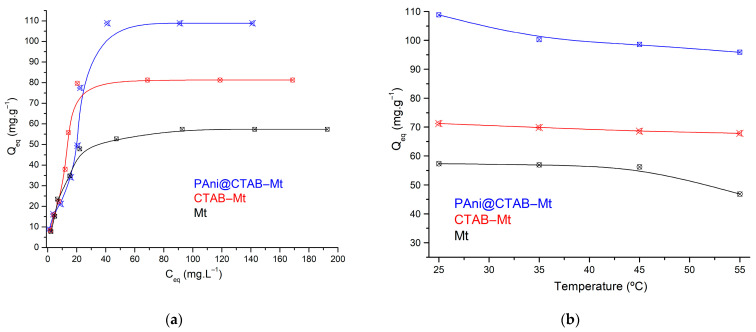
(**a**) Adsorption isotherms; (**b**) temperature influence on removal percentage (adsorbent dose: 0.5 g; MB: 150 mL; T: 298 K; pH: 6.8).

To study the application of the prepared adsorbent, it was necessary to examine the impact of MB dye concentration on the adsorption uptake. Therefore, the initial MB concentration was varied from 10 mg·L^−1^ to 250 mg·L^−1^, and the adsorption uptake of MB on the adsorbent was investigated and reported. The interaction between the adsorbate and adsorbent can be explained using isotherm models. Different equilibrium models were proposed and employed in this research. The collected results were fitted to the Langmuir, Freundlich and Temkin isotherm models in order to find an equilibrium model that best explains the experimental results of this study. The studied isotherm models and the obtained experimental results for the MB removal by PAni@CTAB-Mt are illustrated in Figure 8a. The results show that the MB uptake progressively increased as the initial dye concentration increased and reached saturation with the maximum adsorption capacity of 108.82 mg·g^−1^. In addition, the highest adsorption capacities of 81.25 mg·g^−1^ and 57.36 mg·g^−1^ were achieved by CTAB-Mt and Mt, respectively, at an MB dye concentration of 150 mg·L^−1^. The reason for this trend could be that the initial dye concentration provided a considerable driving force to reduce the resistance of mass transfer between the aqueous and solid phases, and the equilibrium was achieved by introducing of the dynamic balance between the dye concentration and the adsorbent surface [21,22]. Interestingly, the removal capacity of PAni@CTAB-Mt was almost the highest among the adsorbents reported in previous studies for MB (Table 5). Therefore, PAni@CTAB-Mt can be an efficient removal and promising adsorbent material.

The obtained results of the isotherms studied are listed in Table 6. The results demonstrate that the Freundlich isotherm had the highest correlation coefficient (*R*^2^) for all adsorbents. The results also show that the Langmuir model was unable to fit the experimental results, especially when PAni@CTAB-Mt and CTAB-Mt were used as adsorbents. A distinctive feature of the Temkin isotherm model is the uniform distribution of the binding energies. According to the Temkin model, the heat of adsorption for each adsorbate in the layer decreases continuously with coverage and is based on indirect adsorbent/adsorbate interaction [15]. The *R*^2^ values obtained by this model were also significant.

#### 3.2.4. Adsorption Temperature and Thermodynamics

**Figure 9:**
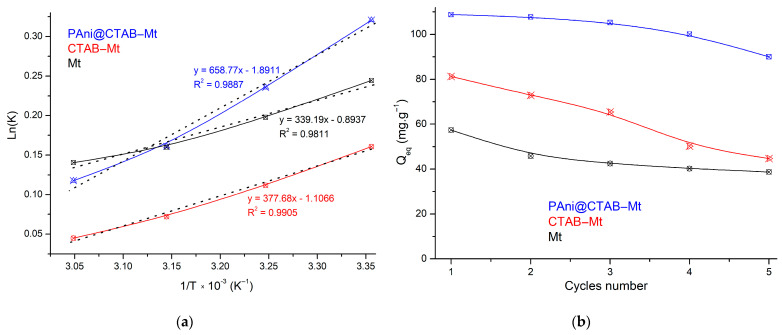
(**a**) Van’t Hoff plot for MB dye adsorption onto adsorbents; (**b**) reusability performance of adsorbents for the adsorptive removal of MB dye (C_0_: 150 mg·L^−1^; T: 298°C; t = 3 h; pH: 6.8).

To study the application of the prepared adsorbent, it was necessary to examine the effect of temperature on the MB elimination by adsorbents; the thermodynamic data of the adsorbents were studied at different temperatures (Figure 8b). The values of ΔS° and ΔH° were determined using the intercept and slope of the Van’t Hoff graph (Figure 9a). The findings of the thermodynamic investigations are listed in Table 7. The resulting values for ΔH° are negative, indicating that the adsorption process is exothermic, and the adsorbent was more efficient at the lower temperatures. In addition, the negative values of the ΔG° show that the adsorption process is spontaneous and feasible. Meanwhile, the ΔG° values increased as the temperature increased, indicating that the adsorption process is more advantageous at room temperature than at higher temperatures. The positive value of ΔS° shows that the entropy has increased because of the MB adsorption on the surface of the adsorbent. In addition, the negative values of ΔS° reveal that the randomness in the system decreases because of the solid–liquid interaction in the adsorption process.

### 3.3. Recyclability of Adsorbents

In order to evaluate the capability of these adsorbents for practical applications, five consecutive cycles of MB adsorption were carried out using the three samples (Figure 9b). It was found that the adsorption capacity of PAni@CTAB-Mt decreased by 4.43% after three cycles of utilizing the adsorbents, which is considered to be an insignificant loss of activity. After the fifth adsorption–desorption cycle, the adsorption capacity decreases by 17.23%. Moreover, the results show that CTAB-Mt and Mt were reduced by 45.14% and 32.83% respectively, after the five cycles. The decreases are probably due to the weakening of the electrostatic interactions caused by the continuous cycling. Therefore, the PAni matrix used in the preparation of modified Mt shows remarkable stability and reusability for MB dye adsorption.

## 4. Conclusions

The test findings in this study show that the PAni@CTAB-Mt adsorbent has an exceptional ability to adsorb MB dye from aqueous solution. A number of variables, including pH, contact time and temperature, were shown to affect the adsorption of MB on these adsorbents. It was expected that the synergy between PAni and CTAB-Mt would impart promising properties to the hybrid adsorbent, as a high amount of MB dye (108.82 mg·g^−1^) was adsorbed on PAni@CTAB-Mt compared to that adsorbed for CTAB-Mt (71.20 mg·g^−1^) and Mt (57.36 mg·g^−1^). The enhanced adsorption capacity of the hybrid adsorbent is attributed to the increase in surface area and pore volume of the hybrid materials. The adsorption followed PSO kinetics, with *R*^2^ values of 0.954, 0.942 and 0.958 for Mt, CTAB-Mt and PAni@CTAB-Mt adsorbents, respectively. The ΔG°, ΔH° and ΔS° at room temperature were found to be −0.794 kJ·mol^−1^, −5.50 kJ·mol^−1^ and −15.79 J·mol^−1^, respectively, for PAni@CTAB-Mt; −0.398 kJ·mol^−1^, −3.14 kJ·mol^−1^ and −9.20 J·mol^−1^, respectively, for CTAB-Mt; and −0.606 kJ·mol^−1^, −2.82 kJ·mol^−1^ and −7.43 J·mol^−^, respectively, for Mt. These results indicate the spontaneous and exothermic nature of the adsorption process. The Freundlich isotherm model has the highest correlation coefficients (*R*^2^), indicating its best suitability to explain the adsorption of MB on homogeneous and heterogeneous surfaces. Furthermore, the desorption and reusability experiment indicated that PAni@CTAB-Mt has the potential to be a reusable adsorbent for MB removal. In general, PAni@CTAB-Mt is an attractive candidate for the removal of dyes from various types of wastewater.

## Figures and Tables

**Table 1:** Elementary chemical composition in weight percent (wt %) of samples.

Weight wt %	SiO_2_	Al_2_O_3_	Fe_2_O_3_	MgO	Na_2_O	K_2_O	TiO_2_	OMC *
Mt	62.61	31.52	1.87	2.03	0.82	0.94	0.20	0.01
CTAB-Mt	56.84	25.12	2.13	1.89	1.98	1.07	0.17	10.80
PANI@CTAB-Mt	54.47	24.79	2.21	1.94	1.56	0.88	0.09	14.06

* OMC: Organic matter content.

**Table 2:** Pore characteristics of the materials.

Materials	PANI	Mt	CTAB-Mt	PANI@CTAB-Mt
S_BET_/m^2^·g^−1^	31	91	73	121
V_DR_ (N_2_)/cm^3^·g^−1^	1.11	0.92	1.37	1.24
V_mes_/cm^3^·g^−1^	0.01	0.01	0.09	0.08
V_mic_/cm^3^·g^−1^	0.01	0.02	0.15	0.13
V_tot_ pore	0.02	0.03	0.24	0.21

**Table 3:** Summary of the XPS analysis obtained for the prepared samples.

Materials	Spectrum	Peak BE * (eV)	Atomic %	Assignments
CTAB-Mt	N1s	399.42	61.11	–NH–
		400.75	38.89	=NH–
	C1s	284.61	62.22	C–C, C–H
		286.08	29.54	O–C=O, C=N, C–N
		287.93	8.24	C–O–C, C=N^+^
PAni@CTAB-Mt	N1s	399.20	56.89	–NH–
		400.24	43.11	=NH–
	C1s	284.56	68.19	C–C, C–H
		286.25	26.88	O–C=O, C=N, C–N
		288.04	4.93	C–O–C, C=N^+^

BE *: Binding Energy.

**Table 4:** PFO, PSO and ID models for MB removal by means of adsorbents.

Model	Constant	Mt	CTAB-Mt	PAni@CTAB-Mt
PFO	$k_{1}$ (min^−1^)	0.033	0.031	0.047
	$q_{eq.Cal}$ (mg·g^−1^)	44.38	68.91	74.47
	*R* ^2^	0.625	0.763	0.599
PSO	$q_{eq.Exp}$ (mg·g^−1^)	69.82	81.25	108.82
	$k_{2ads}$ (g·mg^−1^·min^−1^)	0.0162	0.0035	0.0071
	$q_{eq.Cal}$ (mg·g^−1^)	48.35	52.51	96.50
	*R* ^2^	0.954	0.942	0.958
Intraparticle diffusion	$k_{i}$ (g·mg^−1^·min^−1^)	0.978	1.545	1.332
	*C* (mg·g^−1^)	28.92	37.85	71.14
	*R* ^2^	0.709	0.676	0.678

**Table 5:** Adsorption efficiencies of MB dye on different adsorbents.

Adsorbents	$q_{eq}$ (mg·g^−1^)	$C_{0}$ (mg·L^−1^)	Ref.
PAni@ZnO-SiO_2_	71.20	100	[8]
Activated carbon/cellulose	103.66	100	[23]
Ruthenium nanoparticle loaded activated carbon	94.60	80	[24]
Wheat straw biochar powder	62.50	10	[25]
Algerian palygorskite powder	57.5	10	[26]
Tannin-immobilized cellulose microspheres	55.4	1500	[27]
Cortaderia selloana flower spikes	47.9	50	[28]
Natural Saudi Red Clay	50.25	100	[29]
Natural clay Portugal	22.20	100	[30]
Epoxy/clay nanocomposites	1.424	2000	[31]
Porous geopolymer based pyrophyllite (PyGP4)	64.10	2400	[32]
Geopolymer/ZnTiO_3_/TiO_2_ (GTA)	61.96	150	[33]
Lolium multiforum (LM)	28.70	150	[34]
Treated NaOH/Lolium multiforum (NaOH/LM)	67.19	150	[34]
Treated H_3_PO_4_ 40%/Lolium multiforum (R-40)	80.79	150	[35]
Treated H_3_PO_4_ 70%/Lolium multiforum (R-70)	70.21	150	[35]
Pyrolysis R-40 (AC-40)	8.20	150	[35]
Pyrolysis R-40 (AC-70)	53.32	150	[35]
Citric acid/Kenaf core fiber	144.1	150	[36]
Untreated (Metroxylon sagu) waste	64.21	150	[37]
Potassium hydroxide/(Metroxylon sagu) waste	66.11	150	[37]
Phosphoric acid/(Metroxylon sagu) waste	36.00	150	[37]
Sulphuric acid/Parthenium carbon (SWC)	16.80	150	[38]
Phosphoric acid/Parthenium carbon (PWC)	26.10	150	[38]
Needles of Pinus sylvestris	92.52	150	[39]
PANI@ZnO	59.23	150	[40]
Mt	57.36	150	This work
CTAB-Mt	81.25	150	This work
PAni@CTAB-Mt	108.82	150	This work

**Table 6:** Langmuir, Freundlich and Temkin isotherm parameters for MB removal by adsorbents.

Model	Constant	Mt	CTAB-Mt	PAni@CTAB-Mt
Langmuir	$q_{eq.Exp}$ (mg·g^−1^)	57.36	81.25	108.82
	$q_{eq.Cal}$ (mg·g^−1^)	80.64	12.50	67.56
	$K_{L}$ (L·mg^−1^)	0.098	0.058	0.013
	$R_{L}$	0.112	0.730	0.532
	*R* ^2^	0.806	0.567	0.559
Freundlich	$K_{F}$ (mg^1−1/n^L^1/n^g^−1^)	1.022	1.238	1.584
	n	1.182	1.038	1.398
	*R* ^2^	0.989	0.993	0.972
Temkin	B (T·mol^−1^)	16.99	17.12	11.65
	$K_{T}$ (L·g^−1^)	1.633	1.378	2.627
	*R* ^2^	0.935	0.892	0.861

**Table 7:** Thermodynamic parameters for MB dye adsorption by adsorbents.

Adsorbents	T/K	ΔG/kJ·mol^−1^	ΔH/kJ·mol^−1^	ΔS/J·mol^−1^
Mt	298	−0.606	−2.82	−7.43
	308	−0.531		
	318	−0.457		
	328	−0.383		
CTAB-Mt	298	−0.398	−3.14	−9.20
	308	−0.306		
	318	−0.214		
	328	−0.122		
PAni@CTAB-Mt	298	−0.794	−5.50	−15.79
	308	−0.637		
	318	−0.478		
	328	−0.321		

## Data Availability

Data will be made available on reasonable request.
